# ADRB3 expression in tumor cells is a poor prognostic factor and promotes proliferation in non-small cell lung carcinoma

**DOI:** 10.1007/s00262-020-02627-3

**Published:** 2020-06-08

**Authors:** Meng Zheng, Zhiling Zhou, Xiangting Tian, Dingzhang Xiao, Xinghua Hou, Zhi Xie, Haidan Liang, Shuguang Lin

**Affiliations:** 1 Guangdong General Hospital and Guangdong Academy of Medical Sciences, Guangzhou, China; 2 Zhuhai People’s Hospital, Zhuhai Hospital Affiliated with Jinan University, Zhuhai, China

**Keywords:** ADRB3, Macrophage, Lung cancer, Inflammation, Monoclonal antibody, mTOR

## Abstract

The cross-talk between cancer cells and monocyte-derived alveolar macrophages (Mo-AMs) promotes non-small cell lung carcinoma (NSCLC) progression. In this study, we report that both cancer cells and Mo-AMs robustly express beta 3-adrenergic receptor (ADRB3) in NSCLC. ADRB3 supports lung cancer cells proliferation and promotes chronic inflammation. Genetic and pharmacologic inhibition of ADRB3 reverses tumor growth and inflammation in mouse. Furthermore, we demonstrate that M5D1, a novel anti-ADRB3 monoclonal antibody, inhibits human lung cancer cells proliferation and inflammation via affecting the intracellular mTOR pathway and activating p53. In NSCLC patients, we confirmed that upregulation of ADRB3 expression correlates with tumor progression and poor prognosis. Altogether, these results shed light on the role of ADRB3 in NSCLC and suggest that M5D1 could become powerful antitumor weapons.

**Electronic supplementary material:**

The online version of this article (10.1007/s00262-020-02627-3) contains supplementary material, which is available to authorized users.

## Introduction

The number of deaths from lung cancer is higher than the number of deaths caused by breast, colon and prostate cancers [1]. NSCLC constitutes over 80% of all lung cancer cases [2]. This group includes mainly squamous cell carcinoma (SCC) and adenocarcinoma (AC) [3]. The latest method of immunotherapy uses monoclonal antibodies directed against the immune-checkpoint molecules such as programmed cell death 1 (PD-1) or its ligand (PD-L1) [4, 5]. Cancer progression is affected significantly by immune system dysfunctions, particularly those that involve generalized immunosuppression and the activation of proinflammatory cell pathways [6]. Cancer-related inflammation plays a critical role in facilitating lung cancer growth, invasion and metastasis. However, checkpoint inhibitors PD-1/PD-L1 antibody can cause systemic inflammation that may potentially lead to tumor progression [7]. Inflammation also affects immune surveillance and responses to immunotherapy [8]. Macrophage is one of the most abundant immune cells in the tumor microenvironment of solid tumors. Furthermore, macrophages express cytokines that can suppress antitumor immunity and promote tumor progression [9, 10].

Accumulating evidence suggests that stress-induced sympathetic nervous system (SNS) activation of beta-adrenergic receptor signaling may play a role in the regulation of various cancer types. The ADRB3, a member of G-protein-coupled receptor family, is expressed mainly in adipose tissue and is thought to contribute to lipolysis and thermogenesis [11]. In addition, the overexpression of ADRB3 has been found in several cancer types, including breast cancer [12], gallbladder cancer [13], colorectal cancer [14]. Actually, in recent years, exciting new discoveries have shed new light on the role of ADBR3 in promoting tumor progression, and some important studies deserve to be mentioned. Such studies demonstrate the involvement of ADBR3 in prostate cancer [15] and melanoma [16, 17]. More recently, the involvement of ADRB3 in metabolic reprogramming of melanoma [18], the promotion of immune-tolerance [19], and in regulation of cancer differentiation [20] has been described. However, the detailed clinicopathological analysis of ADRB3 expression in NSCLC has not yet been performed.

Here, we first reported that the expression of ADRB3 was related to an unfavorable patient outcome in NSCLC. We found elevated levels of ADRB3 in lung cancer cells as well as Mo-AMs in the peritumoral region from NSCLC patients. Furthermore, ADRB3 deletion was able to reduce inflammation in the lung of mice by both decreasing lung CD68^+^ macrophages and circulating monocytes associated with anticancer enhancement. Finally, Anti-ADRB3 monoclonal antibody inhibited the growth of lung cancer in mice. This approach could help reduce inflammatory cytokines such as interleukin-6 (IL-6), and contribute to the enhancement of the differentiation of effector T cells in spleen.

## Materials and methods

### Reagents

DMEM/F12 medium and fetal bovine serum (FBS) were from Invitrogen (Carlsbad, CA). ADRB3 antagonist SR59230A (SR) was from Sigma-Aldrich (St Louis, MO). Antibodies against the following proteins were used: ADRB3, Myeloperoxidase (MPO), IL-6, IFN-γ, CD19, CD68, Nucleolin, which were purchased from Abcam (Cambridge, UK); Ki-67, mTOR, Rictor, p53 and GAPDH, which were purchased from Cell Signaling Technology (Danvers, USA).

### Production of mouse ADRB3 monoclonal antibody

A Balb/c mouse was intraperitoneally immunized with human recombinant ADRB3 protein two times at 2-week intervals. Mouse was sacrificed and its spleen cells were collected and fused with myeloma cells by modified hybridoma technique. After the HAT medium (Sigma–Aldrich) selection, the culture supernatants obtained from the hybridoma-containing wells were analyzed for antibody reactivity by Western blotting. A hybridoma cell line was selected and amplified. Balb/c mice received intraperitoneal injection of 10^6^ hybridoma cells, and 8 days after inoculation, the ascites was collected. The saturated ammonium sulfate was concentrated in ascites and purified by Protein A affinity chromatography column.

### Lung tissue arrays, marrow and blood smears

The lung cancer tissue arrays and patient profiles were from Shanghai Outdo Biotech (Shanghai, China). All the patients were diagnosed with primary non-metastatic lung cancer by pathology and did not receive chemotherapy or radiotherapy before surgery. Tissue arrays were constructed from formalin-fixed paraffin-embedded tissue blocks of pretreatment biopsy specimens. Marrow and blood smear of NSCLC patient and healthy person were from bone marrow test lab in Guangdong General Hospital.

### Immunohistochemistry (IHC)

Standard deparaffinization, rehydration and antigen retrieval procedures were performed. Tissue arrays were stained with ADRB3 antibody at dilution of 1:200 for 2 h. HRP activity was visualized using the Liquid DAB Plus Substrate Kit (Thermo Scientific) according to the manufacturer's instructions. At each timepoint, 6 ~ 8 fields were randomly observed and if > 5% of the cells were positive, the section was identified as a case of positive staining. Light yellow brown staining was considered weakly positive (+), brown staining was considered positive (++) and dark brown staining was regarded as strongly positive (+++). The IHC score was interpreted by histopathological evaluation by the authors (MZ and ZX), using the following criteria: 0 = negative; 1 = equivocal/uninterpretable; 2 = weak positive; 3 = strong positive.

### Cell culture and immunofluorescence

The human lung cancer cell lines, A549 were maintained in DMEM/F12 medium containing 10% FBS. Cells were fixed with 4% paraformaldehyde, and permeabilized with 0.1% Triton X-100. Cells were blocked in PBS containing 2% BSA and incubated with mouse anti-Ki67 and rabbit anti-ADRB3 (1:200) overnight at 4℃. Cells incubated with goat anti-mouse secondary antibody conjugated to Alexa Fluor 488 and goat anti-rabbit secondary antibody conjugated to Alexa Fluor 555, followed by DAPI staining. Images were acquired on a scanning confocal microscope (Leica, Germany) and analyzed with Fluorchem 8900 software (Alpha Innotech, CA). ADRB3 expression levels were expressed as the geometric mean fluorescence intensity (MFI).

### Transient transfection with plasmids or siRNAs

pcDNA3-ADRB3 were built. A small interfering RNA (siRNA) for the specific inhibition of ADRB3 expression and a negative control siRNA were synthesized by Sigma (Shanghai, China). A549 cells were plated in a 6-well plate before transfection. After 70% confluence was achieved, 2 μg plasmids or 100 pmol siRNA was diluted with 100 μL serum-free medium using the Lipofectamine 3000 transfection reagent (Life Technologies, CA) according to the manufacturer’s instructions.

### MTT assay

For the MTT assay, 200 μl of 0.5 mg/ml MTT-tetrazolium salts (Sigma–Aldrich) in DMEM/F12 was added to each well. After 4 h of incubation, the formazan crystals were dissolved by adding DMSO. The absorption of the formazan solution was measured using Multiskan G0 spectrophotometer at a wavelength of 570 nm.

### Cell cycle analysis

For flow cytometric tests of the cell cycle, propidium iodide (PI) staining was measured at an excitation wavelength of 488 nm and emission wavelength of 670 nm.

### Western blot analysis

Whole-cell lysates were generated using RIPA lysis buffer (Abcam, UK). Total proteins were separated using 10% SDS-PAGE and then transferred onto a nitrocellulose membrane. The membrane was incubated with the primary antibody at 4 °C overnight, followed by a horseradish peroxidase-conjugated secondary antibody the next day for 1 h at room temperature. The immunoreactive bands were visualized using enhanced chemiluminescence reagents.

### Real-time PCR

RNeasy Mini Kit was used (Qiagen, Germany) for RNA isolation. The reverse transcription reaction was performed using High-Capacity cDNA Reverse Transcription Kit with RNase Inhibitor (Applied Biosystems, USA). Changes in the expression level of ADRB3 were tested using ABI 7500 real-time PCR system. The results were standardized, based on the expression of the reference gene of β-actin. The obtained results were shown in the graphs on a logarithmic scale and subjected to statistical analysis. Mouse ADRB3-forward primer: 5′-GACAGCCTCAAATGCATCCT-3′; Mouse ADRB3-reverse primer: 5′-CCCAGTCCACACACACCTTTCT-3′.

### Mice

FVB/N genetic background ADRB3-null mice were purchased from JAX. C57BL/6 genetic background ADRB3-null mice were generated using C57BL/6 mice backcrossed for nine generations. All mice used in this study were maintained and used at the Guangdong General Hospital mouse facility under pathogen-free conditions according to institutional guidelines and animal study proposals approved by the Institutional Animal Care and Use Committee.

### Tumor implantation

C57BL/6 mice were injected with 2 × 10^5^ LLC cells obtained from the China Type Culture Collection. Tumor dimensions were calculated by caliper measurements and volume was calculated according to the equation *V* = (*π*/6) × *a* × *b* × *c,* where *a*, *b* and *c* are diameters in three perpendicular dimensions.

### Multiplex ELISA array

Plasma levels of IL-2, IL-5 and IL-6 were determined using the Mouse Th1/Th2 Array Q1 Kits (RayBiotech, GA). MPO was determined using the Mouse MPO Quantikine ELISA Kits (R&D Systems,) according to manufacturer's instructions.

### Electrophoretic mobility shift assay (EMSA)

An EMSA was performed using oligos corresponding to p53 (5′‐TACAGAACATGTCTAAGCATGCTGGGGACT‐3′) as biotin-labeled probe. Nuclear proteins were extracted using Nuclear and Cytoplasmic Protein Extraction Kit (Viagene, China). For each sample, 6 μg nuclear proteins were pre-incubated with the Gel-Shift Binding Buffer to block non-specific binding prior to the addition of the probe (100 pmol) and further incubation for 20 min at 25 °C. For supershifts, nuclear extracts were pre-incubated with 3 μg p53 antibody or non-immune IgG (Santa Cruz Biotechnology) (negative control) for 15 min at 4 °C prior to addition of the probe. Electrophoresis was carried out on non-denaturating polyacrylamide gels (6%) in 0.5 × TBE at 100 V for 90 min and then electrophoretically transferred onto a positively-charged nylon membrane in 0.5 × TBE at 300 mA for 45 min. The DNA–protein complex was visualized with streptavidin-HRP Conjugate.

### Statistics

Statistical analysis was performed with SPSS 21.0 software (IBM, NY). Continuous variables were expressed as means ± SEM. Categorical variables were compared by Chi square test. The Kaplan–Meier method was used to evaluate the survival of patients. The log-rank test was used to evaluate the differences between groups. *P* < 0.05 was considered statistically significant.

## Results

### High expression of ADRB3 on cancer cells and alveolar macrophages (AMs) predicts poor prognosis in NSCLC

**Fig. 1:**
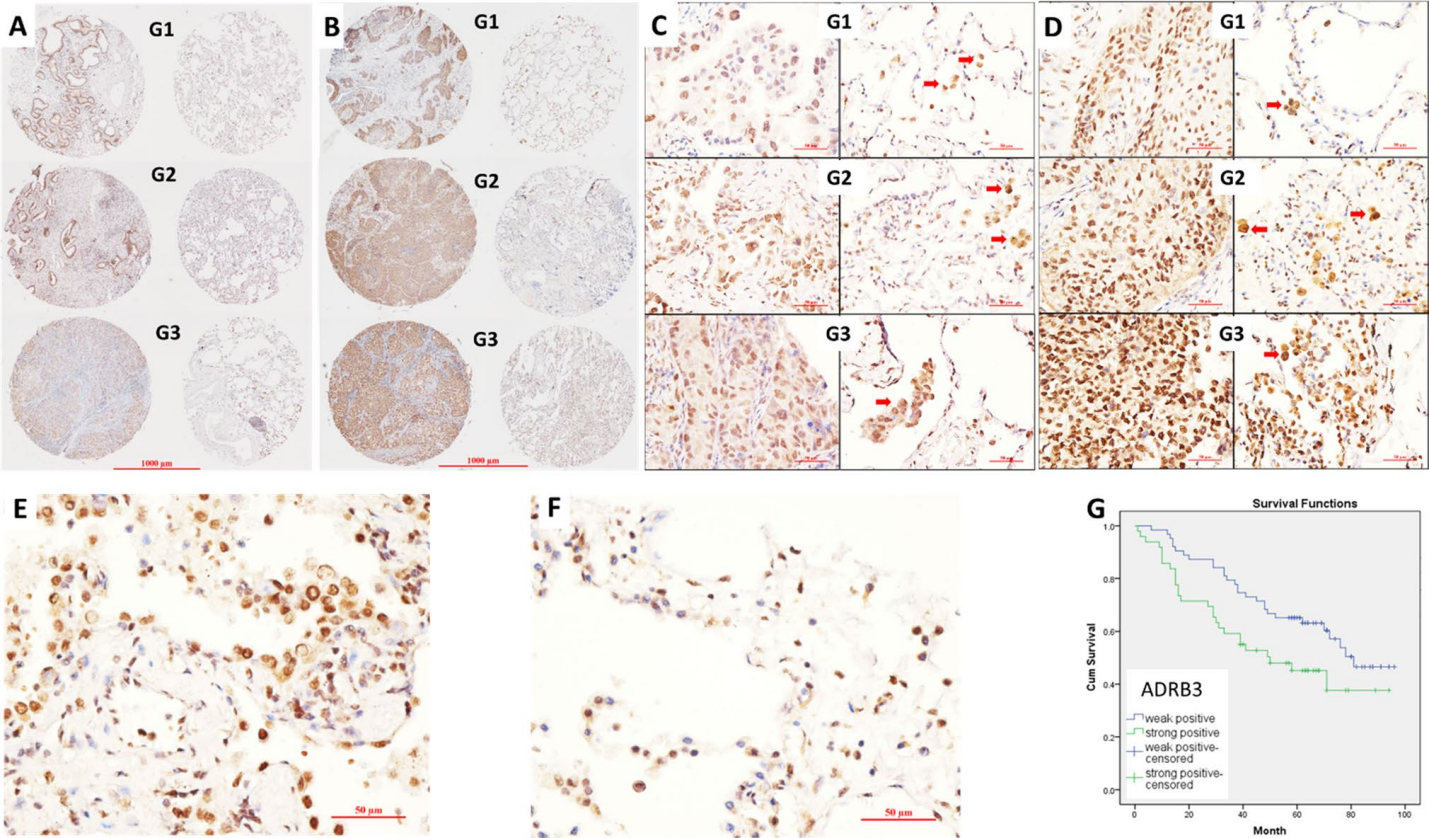
ADRB3 expression in tumor cells and AMs in NSCLC patients. **a** Representative tissue spots of ADRB3 expression in cancerous tissue and paracancerous tissue of different grade G of AC patients. Each row represents two specimen from the same patient; the left is cancerous tissue, the right is paracancerous tissue. **b** Representative tissue spots of ADRB3 expression in cancerous tissue and paracancerous tissue of different grade of SCC patients. Each row represents two specimen from the same patient; the left is cancerous tissue, the right is paracancerous tissue. **c** Representative IHC staining of ADRB3 in cancerous tissue and paracancerous tissue of different grade of AC patients. Each row represents two specimen from the same patient; the left is cancerous tissue, the right is paracancerous tissue. Red arrow points to the AMs. **d** Representative IHC staining of ADRB3 in cancerous tissue and paracancerous tissue of different grade of SCC patients. Each row represents two specimen from the same patient; the left is cancerous tissue, the right is paracancerous tissue. Red arrow points to the AMs. **e** Representative IHC staining of ADRB3 in squamous dysplasia. **f** Representative IHC staining of ADRB3 in paracancerous tissue with inflammatory cell infiltration. **g** Comparison of Kaplan–Meier curves presenting overall survival percentage in patients with NSCLC according to weakly and strongly levels of ADRB3 expression. Magnification (**a**, **b**) ×40, magnification (**c**, **d**, **e**, **f**) ×400

To examine the distribution of ADRB3 expression in NSCLC, we performed ADRB3 immunostaining on NSCLC tissue microarray (TMA). ADRB3 was only expressed in AMs of paracancerous tissue. In contrast, both cancer cells and AMs expressed ADRB3 at higher level in cancerous tissue (Fig. 1a–d). Moreover, ADRB3 was also highly expressed in squamous dysplasia (Fig. 1e).

ADRB3 was localized in the cytoplasm and nucleus of tumor cells. The positive expression rate of ADRB3 in cancerous tissue (94.6%) was markedly higher than that in paracancerous tissue (7.2%) of 166 NSCLC samples including 87 AD and 79 SCC (Table 1). There is no statistical difference of ADRB3 expression between AD and SCC groups. 57 (34.3%) cases had positive staining in ≥ 25% tumor cells (TC). 37 (22.3%) cases had positive staining in ≥ 50% TC. The higher grade **g** of malignancy, the higher ADRB3 expression was observed. The differences were statistically significant between G1 versus G2 and G1 versus G3 (Table 1). ADRB3 expression was increased in higher stage (S) of NSCLC. The differences were statistically significant between S1 versus S2 and S1 versus S3 (Table 1). In addition, the expression of ADRB3 in AMs was higher from the patients with inflammatory cell infiltration (ICI) (Fig. 1f, IHC score, 2.7 ± 0.4) compared to patients without ICI (IHC score, 1.8 ± 0.3, *P* < 0.05). Finally, we evaluated the association between ADRB3 expression in lung cancers and the impact on patients’ survival. Log-rank (Mantel-Cox) analysis showed that patients with weak-positive ADRB3 had longer survival compared to the group with strong-positive (*P* = 0.038, Fig. 1g).

**Table 1:** Correlations between the expression of ADRB3 and clinicopathological parameters

	*n*	ADRB3 positive	ADRB3 (2–3 points)	*P*
Cancer tissue	166	157 (94.6%)	94 (56.6%)	Compared with cancer tissue,^*^ χ^2^ = 253.4, *P* < 0.001.^#^*χ*^2^ = 127.5, *P* < 0.001
Paracancerous tissue	166	12 (7.2%^*^)	1 (0.6%^#^)	
< 60 years	63	60 (95.2%)	35 (55.6%)	
≥ 60 years	101	95 (94.1%)	59 (58.4%)	
Male	116	109 (94.0%)	65 (56.0%)	
Female	50	48 (96.0%)	29 (58%)	
Grade 1	14	13 (92.9%)	4 (28.6%)	Compared with grade 1, ^△^*χ*^2^ = 4.8, *P* = 0.029; ^△△^*χ*^2^ = 4.2, *P* = 0.040
Grade 2	106	101 (95.3%)	63 (59.4%^△^)	
Grade 3	45	42 (93.3%)	27 (60.0%^△△^)	
Stage I	67	63 (94.0%)	30 (44.8%)	Compared with stage I, ^◇^*χ*^2^ = 5.8, *P* = 0.016; ^◇◇^*χ*^2^ = 4.2, *P* = 0.041
Stage II	31	29 (93.5%)	22 (71.0%^◇^)	
Stage III	57	54 (94.7%)	36 (63.2%^◇◇^)	
Adenocarcinoma	87	83 (95.4%)	47 (54.0%)	
Squamous carcinoma	79	75 (94.9%)	47 (59.5%)	

### ADRB3 promotes A549 cell proliferation and cell-cycle progression

**Fig. 2:**
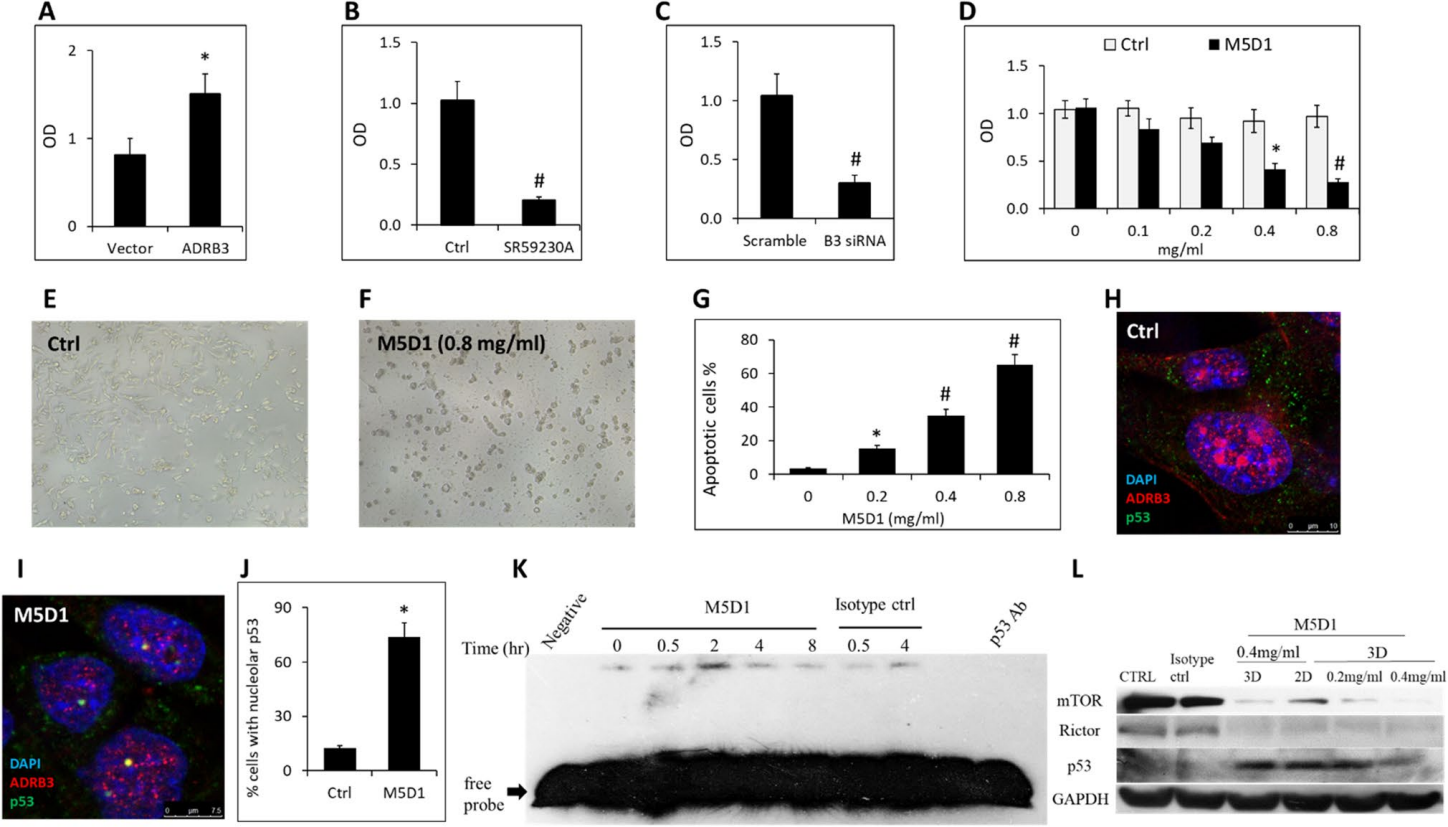
ADRB3 promots the proliferation of A549 cells. **a**, **b**, **c**, **d** The viability of A549 cells modified with pcDNA3-ADRB3, SR59230A, ADRB3 siRNA and anti-ADRB3 monoclonal antibdy (M5D1) were detected by the MTT assay, respectively. **e**, **f** A549 cells were treated with isotype IgG or M5D1 for 96 h. Cell viability was measured by photographed image with a light microscope (magnification, × 200). **g** A549 cells were treated with 0, 0.2, 0.4, 0.8 mg/ml M5D1 for 48 h and apoptotic cells were measured by performing Annexin V/PI staining. The data represent means ± SEM from 5 independent experiments. *P < 0.05; #*P* < 0.01. (H, I) Subcellular localization of p53 (green) and ADRB3 (red) after control or M5D1-treatment of A549 cells. DAPI was used for staining nuclei. All confocal images were captured using 63X oil immersion objective. **j** M5D1-treatment for 12 h induces nucleus accumulation of p53 in 73.8% of A549 cells. **k** M5D1 activated p53 in A549 cells. A549 cells were treated with 0.4 mg/ml M5D1 or control for different time as indicated and p53 activation was determined by EMSA. The nuclear extract of A549 cells were incubated with p53 antibody (PAb421, 200 ng) or 100-fold molar excess of unlabeled oligo to detect supershift and cold competition, respectively. **l** mTOR, Rictor and p53 were determined by western blotting from A549 cells treated with M5D1 for 2 or 3 days

To explore ADRB3 function in lung cancer cells, pcDNA3-ADRB3 was generated and transfected A549 cells. After 96 h, we performed MTT-assay to determine the viability. We found that pcDNA3-ADRB3 conferred a proliferative advantage to A549 cells suggesting a role of ADRB3 in promoting lung cancer growth (Fig. 2a). Conversely, the selective ADRB antagonist SR59230A and ADRB3 siRNA resulted in significant decreases in cell proliferation (Fig. 2b, c). Anti-human ADRB3 monoclonal antibody (M5D1) developed by our team also inhibited A549 cells viability in a dose-dependent way (Fig. 2d–f). In addition, M5D1-treatment resulted in a dose-dependent increase of apoptosis (Fig. 2g).

Relocation of p53 to the nucleus after cellular stress is desirable to inhibit the growth of malignant cells [21]. So we examined p53 localization in A549 cells cultured in the presence of 0.4 mg/ml M5D1 for 12 h. We found that treatment with M5D1 induced p53 nucleus accumulation in 73.8% of the A549 cells (Fig. 2h–j). To test the activity of p53, EMSA was applied. As shown in Fig. 2k, p53 was activated by M5D1 (0.4 mg/ml), peaking at 2 h and declining at 4 h post-treatment. Furthermore, we tested the effect of M5D1 on modulating cell-cycle progression and found G1 arrest from 22.4 ± 3.5% in Isotype IgG to 65.3 ± 7.1% in M5D1 (0.4 mg/ml) cells (*P* < 0.01). We then analyzed the effect of ADRB3 on cell cycle regulators in A549 cells. A strong increase in p53 and decrease in mTORC2 was detected in response to the M5D1 compared with the control (Fig. 2l).

### ADRB3 expression in monocytes is upregulated in NSCLC patients

**Fig. 3:**
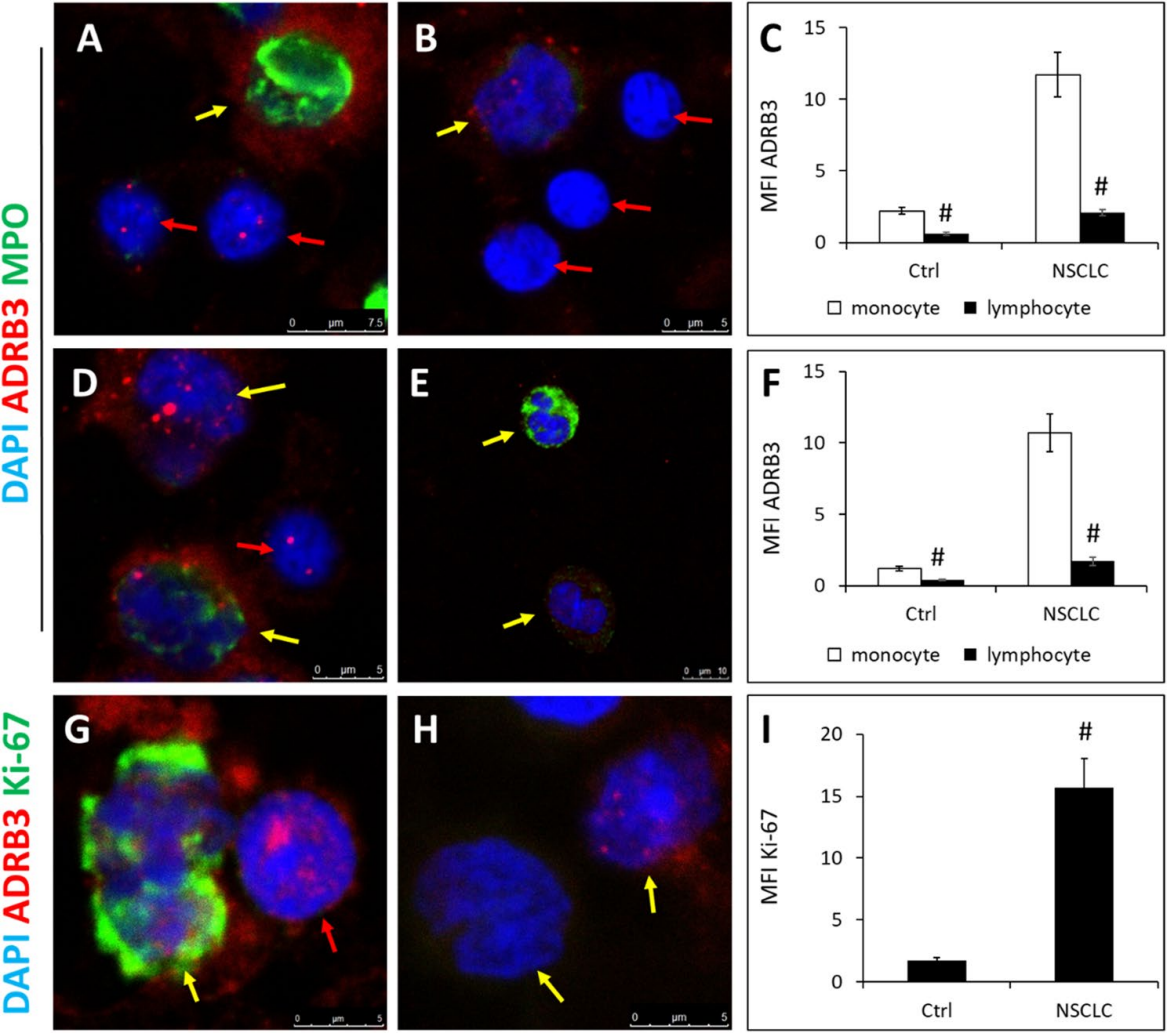
High expression of ADRB3 and Ki-67 in monocytes in NSCLC. **a**, **b** Representative images of ADRB3 expression in myeloperoxidase (MPO) positive monocytes and lymphocytes in bone marrow smear of NSCLC patient and healthy control. Red arrows indicate lymphocytes, yellow arrows indicate monocytes. Cells were co-stained with antibodies against MPO (green) and ADRB3 (red) and DAPI (blue). All images were captured using 63X oil immersion objective. **c** Mean fluorescent intensities (MFI) of ADRB3 in leukocytes in bone marrow of NSCLC patients (*n* = 6) and controls (*n* = 6). All data are expressed as group means ± SEM. #*P* < 0.01. **d**, **e** Representative images of ADRB3 expression in leukocytes in peripheral blood smear of NSCLC patient and controls. **f** MFI of ADRB3 in leukocytes in peripheral blood of NSCLC patients (*n* = 6) and controls (*n* = 6). **g**, **h** Representative images of Ki-67 expression in ADRB3^+^ monocytes in peripheral blood smear of NSCLC patient and healthy control. **i** MFI of Ki-67 in ADRB3^+^ monocytes in peripheral blood of NSCLC patients (*n* = 6) and controls (*n* = 6)

Circulating monocytes are recruited to the lung, where they differentiate into monocyte-derived alveolar macrophages (Mo-AMs), so the immunofluorescence staining of bone marrow, blood and tumor tissue for ADRB3 was performed. ADRB3 expression in monocytes and lymphocytes of bone marrow increased significantly in NSCLC compared with healthy control (MFI 11.7 ± 1.6 versus 2.2 ± 0.6, *P* < 0.01; 2.1 ± 0.2 versus 0.6 ± 0.1, *P* < 0.01, respectively, Fig. 3a–c). High expression level of ADRB3 was observed when we compared NSCLC patient's peripheral blood monocytes and lymphocytes with cells from healthy individuals (MFI 10.7 ± 1.3 versus 1.2 ± 0.2, *P* < 0.01; 1.7 ± 0.3 versus 0.4 ± 0.1, *P* < 0.01, respectively, Fig. 3D-F). Furthermore, we found that Ki-67 expression in ADRB3^+^ monocytes increased significantly in NSCLC compared with healthy control (MFI 15.5 ± 2.3 versus 1.7 ± 0.2, *P* < 0.01, Fig. 3g–i). These data suggests that ADRB3^+^AMs may be Mo-AMs. These observations suggest that ADRB3 is produced by tumor cells and monocytes and plays a critical role in the recruitment of the proliferative monocytes from the blood into tumor tissues, where they differentiate into inflammatory Mo-AMs which interact with tumor cells and are involving in shaping immunosuppressive microenvironment.

### ADRB3 is essential for cancer and inflammation in mice

**Fig. 4:**
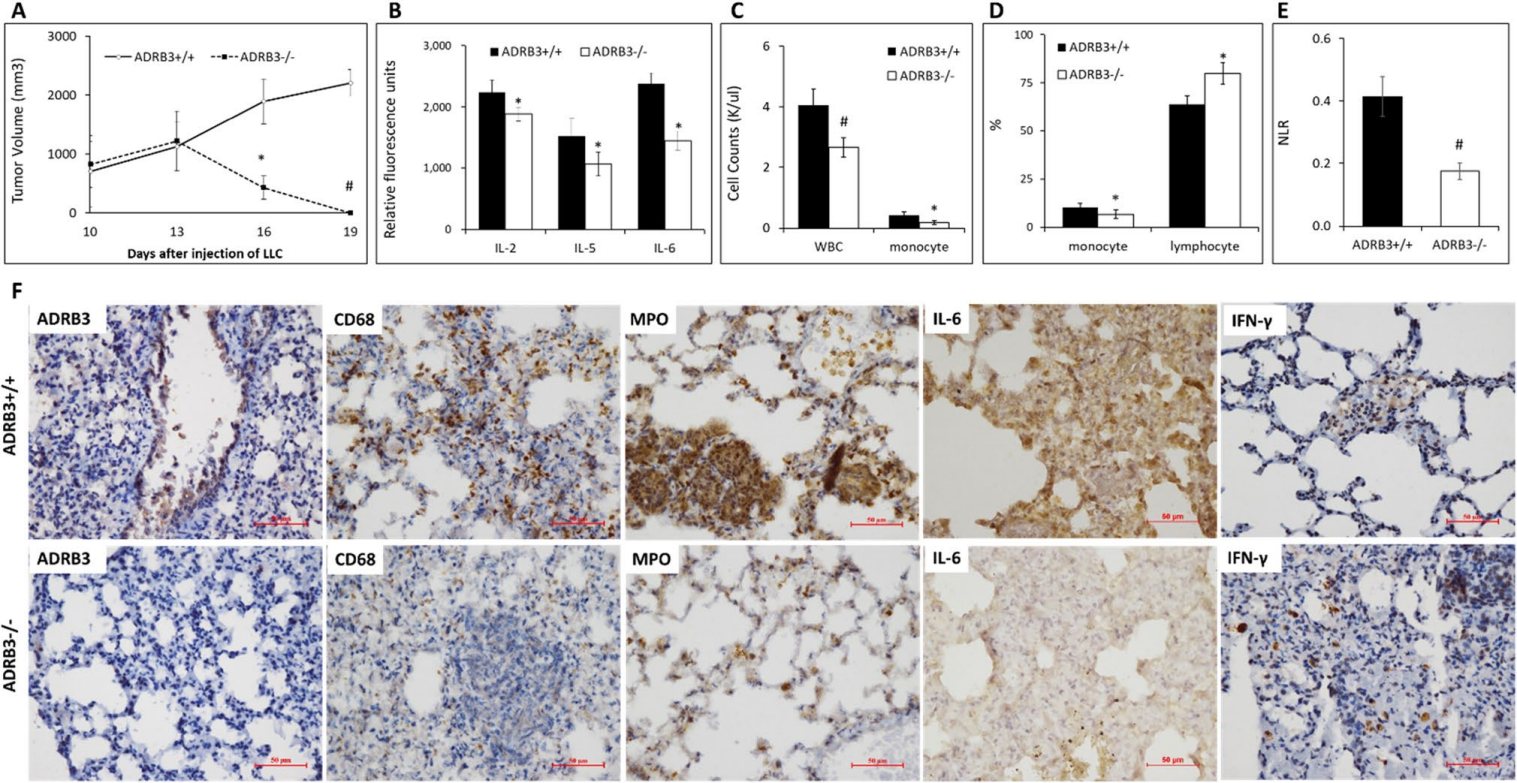
Mice lacking ADRB3 are protected from tumor and inflammation. **a** LLC cancer cells were s.c. inoculated into ADRB3^+/+^ and ADRB3^−/−^ mice (*n* = 8) on day 0. primary tumors were measured with a calipers. *Points*, average diameters of primary tumor as a function of days after tumor inoculation; All data are expressed as group means ± SEM. *P < 0.05, #*P* < 0.01 compared with ADRB3^+/+^ mice. **b** Serum IL-2, IL-5 and IL-6 as measured by Multiplex ELISA Array were significantly decreased in ADRB3^−/−^ mice (*n* = 8) compared with the ADRB3^+/+^ group. **c** Count of white blood cell and monocytes of the ADRB3^−/−^ mice and ADRB3^+/+^ mice. **d** Percentage of monocyte and lymphocytes of the ADRB3^−/−^ mice and ADRB3^+/+^ mice. **e** NLR of the ADRB3^−/−^ mice and ADRB3^+/+^ mice. EDTA anti-coagulated blood samples were used to obtain a complete blood count with automated hematology analyzer. **f** Representative immunostaining of ADRB3, CD68, MPO, IL-6, and IFN-*β* in lung tissues of ADRB3^−/−^ and ADRB3^+/+^ mice. Scale bars, 50 μm

Based on ADRB3 expression in cancer cells, Mo-AMs, monocytes and lymphocytes, we wondered whether ADRB3 would modulate tumor immunity in vivo. The role of ADRB3 in tumor immunity was investigated using mouse tumor models with invasive mouse lung carcinoma (LLC, 2 × 10^6^ cells per mouse) established in C57BL/6 background ADRB3^−/−^ (A 306 bp genomic fragment containing the sequences encoding the third through the fifth transmembrane domains was replaced with a neomycin selection cassette) and their ADRB3^+/+^ littermate. During 2 weeks after subcutaneous inoculation of LLC cells, we observed almost equal tumor growth in both ADRB3^−/−^ and ADRB3^+/+^ mice. However, in the subsequent weeks, significant tumor regression was found in ADRB3^−/−^ mice, where the tumors became undetectable at week 3 (Fig. 4a). In contrast, the tumors in ADRB3^+/+^ mice grew rapidly and invasively, resulting in 100% mortality.

The significant decrease in the levels of serum Interleukin-2 (IL-2), IL-5 and IL-6 (Fig. 4b) and decrease in the number of white blood cells, monocytes (Fig. 4c) were found in ADRB3^−/−^ mice compared with ADRB3^+/+^ mice. There was no difference in the number of lymphocytes. The percentage of monocyte was significantly decreased and the percentage of lymphocytes was increased in ADRB3^−/−^ mice (Fig. 4d). Biomarkers of inflammation such as neutrophil–lymphocyte ratio (NLR) (Fig. 4e) was decreased in ADRB3^−/−^ mice.

Macrophages marker CD68, proinflammatory cytokines mainly produced by macrophages such as MPO and IL-6 [22] were all expressed at lower levels in ADRB3^−/−^ mice lung than ADRB3^+/+^ mice (Fig. 4f). The downregulation of CD68, MPO and IL-6 suggests that ADRB3 knockout eliminate recruitment and activation of macrophages in lung. However, the expression of IFN-γ was higher in ADRB3^−/−^ mice lung, suggesting that ADRB3 knockout might promote naive T cell differentiation into T helper type 1 (Th1) effector cells and increased IFN-γ production. The impaired antitumour effect of CD4 + T cells with their defective Th1 differentiation is restored by IL-6 deficiency [23]. It is speculated that ADRB3 knockout enhances Th1 differentiation with downregulation of IL-6.

### A novel ADRB3 monoclonal antibody exerts anti-cancer and anti-inflammation activity

We found that lung cancer cells aberrantly express ADRB3 and activation of this receptor promotes tumor cell growth. ADRB3 is also present on Mo-AMs that help tumors evade immune response. ADRB3 is likely to be a novel promising therapy target for cancer, so we've developed anti-ADRB3 monoclonal antibodies. As in Supplementary Fig. 1, the SDS-PAGE analysis of purification of ascites fluid produced by hybridoma clone M5D1. From the analysis, lane 5, 7, 9 showed the molecular weight of IgG, for heavy chain was ~ 55 kDa and its light chain was ~ 25 kDa. After purification process of antibody, the purify monoclonal antibody M5D1 from ascites fluids was test with immunofluorescence technique to identify antigens expressed on A549 cells. Using dilution at 1:100 of purified antibody the experiment was indicated that a bright of fluorescence labeled on the cytoplasm of A549 cells as in Supplementary Fig. 2.

To examine the efficacy of the M5D1 in inhibiting lung tumor growth in vivo, we utilized xenograft mouse model and evaluated the growth of tumors after injection of the antibody into mice. C57BL/6 mice were subcutaneously injected with LLC cancer cells in the dorsal right flank and allowed to grow until the tumor reached 80 mm^3^. The mice were intravenously injected through the tail vein with 50 μg M5D1 every 5 days or isotype IgG.

**Fig. 5:**
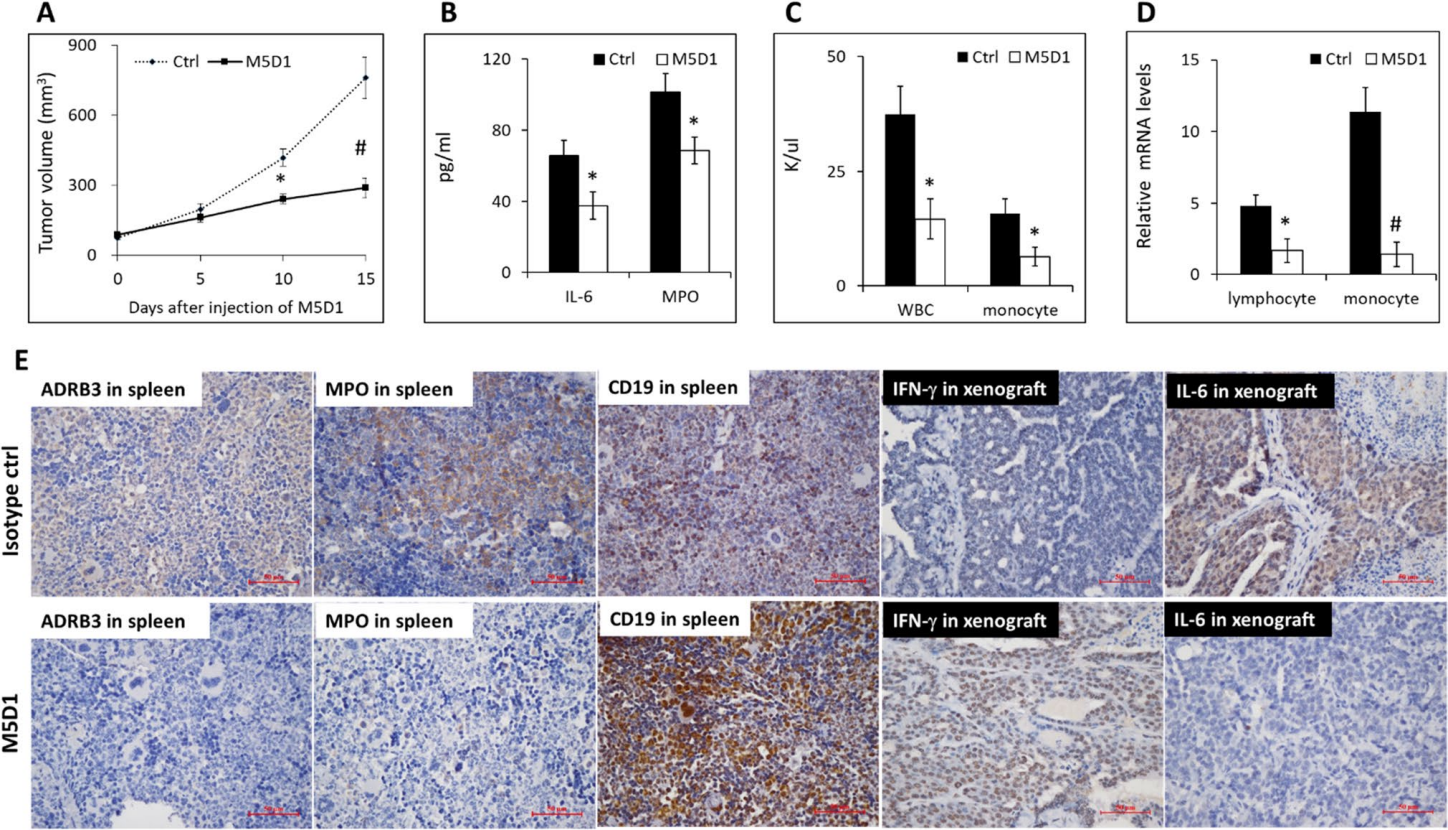
LLC tumor-bearing C57BL/6 mice treated with M5D1 have reduced tumor growth and inflammation. **a** Tumor volumes for each treatment group. **b** Serum IL-6 and MPO as measured by ELISA. **c** The WBC and monocytes count of each group. **d** Results of quantitative real-time PCR are shown as the fold-change in ADRB3 mRNA levels in lymphocytes and monocytes of tumor-bearing mice treated with control IgG or M5D1 compared with age-matched non-tumour-bearing mice, using β-actin as housekeeping gene and compared using 2^−ΔΔCt^ method. All data are expressed as group means ± SEM. *P < 0.05, #*P* < 0.01. **e** Immunostaining of ADRB3, MPO, CD19, IFN-γ and IL-6 in the spleen or tumor, scale bars, 50 μm

When assessing tumor volume, we discovered that mice treated with M5D1 displayed reduced growth of tumors compared to control (Fig. 5a). Furthermore, body weight of the mice was increased by treatment with M5D1. The significant decrease in the level of serum IL-6, MPO (Fig. 5b) and decrease in the number of WBC and monocytes (Fig. 5c) was found in M5D1 group. There was no difference in the number of red blood cells and lymphocytes.

Mice peripheral blood mononuclear cells (PBMCs) were isolated by Ficoll density gradient centrifugation, lymphocytes and monocytes were separated by their adherence to the culture plate. ADRB3 expression was assessed by qPCR analysis. We found that lymphocytes isolated from non-tumor-bearing mice expressed very low to undetectable ADRB3, whereas ADRB3 expression was higher in lymphocytes (4.8-fold; Fig. 5d) and monocytes (11.4-fold; Fig. 5d) from tumor-bearing mice compared with non-tumor-bearing mice. After 15d of M5D1 treatment, the lymphocytes and monocytes from tumor-bearing mice showed an average 1.7- or 1.4-fold increase in ADRB3 expression, respectively, compared with non-tumor-bearing mice.

Immunohistochemistry showed that M5D1 downregulated the expression of ADRB3, MPO, and increased the expression of CD19 in the spleen tissues (Fig. 5e). CD19 regulates B-cell development, activation and differentiation. The upregulation of CD19 suggests that ADRB3 blockade can induce B cells to proliferate and differentiate into CD19^high^ blasts. ADRB3 might be working as a molecular switch, which turns the differentiation signals off in appropriate time, and its absence may leave constitutively activated differentiation-promoting signals on, at least regarding the mTOR pathway. In the tumor tissues, M5D1 significantly decreased IL-6 and increasd IFN-γ (Fig. 5e). IFN-γ is a cytokine produced both by macrophages and by Th1. It is speculated that ADRB3 blockade enhances Th1 differentiation with downregulation of IL-6 and upregulation of IFN-γ. Overall, these results suggested that ADRB3 blockade can enhance the innate and/or adaptive antitumor immune response by dampening macrophage function.

## Discussion

Our study demonstrated that tumor samples exhibited a higher expression pattern of ADRB3 by analyzing NSCLC tissue microarrays. By evaluating the relationship between ADRB3 expression and clinicopathologic parameters, we found that high expression of ADRB3 was strongly correlated with poor tumor differentiation and clinical stage. Our works further demonstrated that patients with higher expression of ADRB3 had a shorter survival time and confirmed that ADRB3 expression is an independent predictor of the overall survival in patients with NSCLC. Then, we found that ADRB3 as an oncogene and demonstrated that ADRB3 attenuates p53-regulated apoptotic pathway in response to stress. Once within the nucleus, ADRB3 regulates the expression of target genes related to proliferation and metastasis, such as mTOR. ADRB3 blockade inhibited lung cancer cell proliferation by inducing p53 nucleus accumulation and subsequently attenuating the expression of mTOR complex 2.

We also found that ADRB3 was highly expressed in Mo-AMs which orchestrate a proinflammatory [24] and profibrotic response [25] and promotes the progression of premalignant lesions such as squamous dysplasia to SCC. These observations suggest that ADRB3 blockade might be an interesting strategy for preventing lung cancer development by inhibiting Mo-AMs.

The level of Ki-67 in ADRB3^+^ monocytes in NSCLC was higher, indicating ADRB3 promotes monocytes exit from the quiescence and re-enter the cell cycle and begin to proliferate. In addition, we found that M5D1 decreases pro-inflammatory cytokine such as IL-6 produced by monocytes. These findings indicate that monocytes in NSCLC are activated by ADRB3 to produce increased amounts of IL-6, which may be one of the mediators involved in the regulation of both local and systemic inflammatory reactions occurring in NSCLC. In addition, IL-6 is a rational immunosuppressive factor in antitumor immune responses through myeloid-derived suppressor cells and T cells [23, 26]. ADRB3 blockade downregulates IL-6 level and monocytes count, these data suggest that ADRB3 attenuates Th1 response partly through enhancing the production of IL-6 from monocytes/macrophages.

Very few effective anti-ADRB3 monoclonal antibodies are clinically available. We successfully developed a novel ADRB3 monoclonal antibody called M5D1, which can targeted kill cancer cells and enhance antitumor immune responses by targeting immune cells. In mice studies, this dual-purpose antibody was highly effective in inhibiting tumors.

In conclusion, our findings shed light on the role of ADRB3 in NSCLC and cancer-related inflammation, thereby highlighting its potential as a therapeutic target.

## Electronic supplementary material

Below is the link to the electronic supplementary material.

Supplementary file1 (PDF 442 kb)

Supplementary file2 (DOCX 13 kb)

## Abbreviations

AC: Adenocarcinoma
ADRB3: Beta 3-adrenergic receptor
AMs: Alveolar macrophages
EMSA: Electrophoretic mobility shift assay
FBS: Fetal bovine serum
ICI: Inflammatory cell infiltration
IHC: Immunohistochemistry
IL-6: Interleukin-6
Mo-AMs: Monocyte-derived alveolar macrophages
MPO: Myeloperoxidase
NLR: Neutrophil–lymphocyte ratio
NSCLC: Non-small cell lung carcinoma
PBMCs: Peripheral blood mononuclear cells
PD-1: Programmed cell death 1
PI: Propidium iodide
SCC: Squamous cell carcinoma
SNS: Sympathetic nervous system
siRNA: Small interfering RNA
TMA: Tissue microarray
TC: Tumor cells
