# Supplementary figures and images for: ADRB3 expression in tumor cells is a poor prognostic factor and promotes proliferation in non-small cell lung carcinoma
Source: Cancer Immunol Immunother. 2020 Jun 8;69(11):2345–55. doi: 10.1007/s00262-020-02627-3 (PMC7568706; doi:10.1007/s00262-020-02627-3)

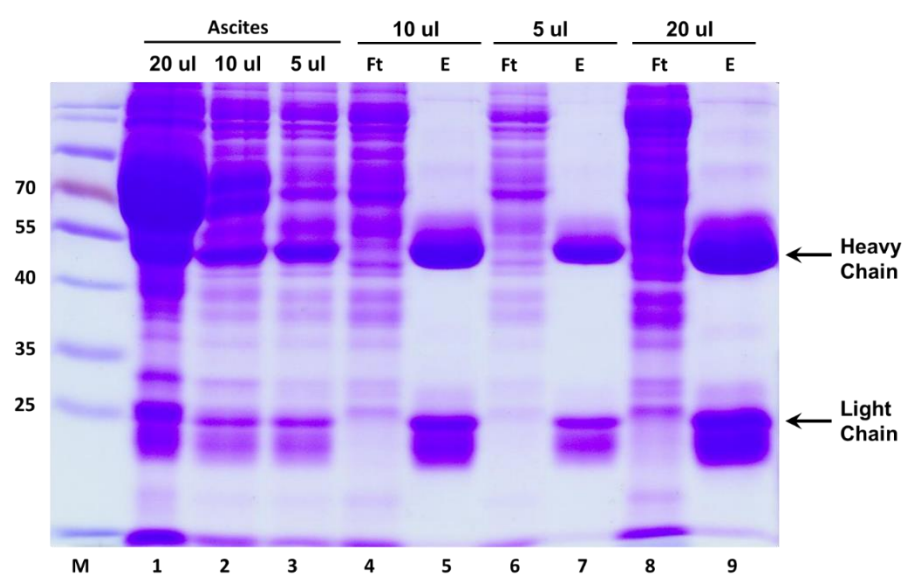

Supplementary Figure 1.

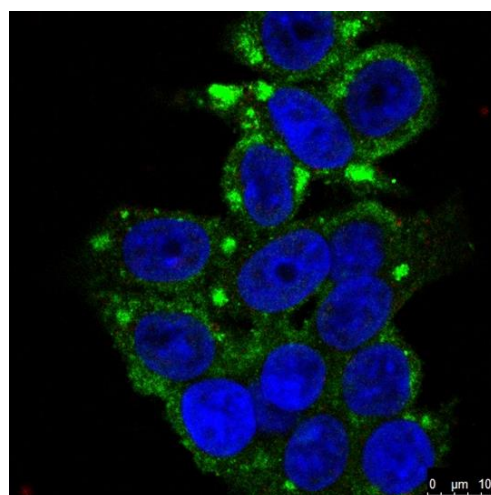

Supplementary Figure 2.

Supplement: Supplementary file 1 — Supplementary file1 (PDF 442 kb) [file 262_2020_2627_MOESM1_ESM.pdf]
