# Supplementary material for: ADRB3 expression in tumor cells is a poor prognostic factor and promotes proliferation in non-small cell lung carcinoma
Source: Cancer Immunol Immunother. 2020 Jun 8;69(11):2345–55. doi: 10.1007/s00262-020-02627-3 (PMC7568706; doi:10.1007/s00262-020-02627-3)
Supplement: Supplementary file 2 — Supplementary file2 (DOCX 13 kb) [file 262_2020_2627_MOESM2_ESM.docx]

**Supplementary Figure 1. Coomassie Blue stained SDS-PAGE analysis for the purification of monoclonal antibody against ADRB3 after affinity chromatography on a 5-ml Protein A column.**

Lane M, Lane M, protein molecular weight marker; Lane 1-3, The dialyzed ascites in PBS after ammonium sulfate precipitation; Lane 4, flow through (Ft) liquid of the ascites; Lane 5, protein eluted (E) from the column; Lane 6, flow through liquid of the ascites; Lane 7, protein eluted from the column; Lane 8, flow through liquid of the ascites; Lane 9, protein eluted from the column.

**Supplementary Figure 2. The immunofluorescence reactivity of purified monoclonal antibody M5D1 features on A549 cells.**

M5D1 was detected with goat anti-mouse (green). Nuclear DNA was labelled with DAPI (blue). Image was taken with confocal microscope using 63X oil immersion objective.
